# Adherence to anti-seizure medications and self-reported availability and affordability of the medications in Addis Ababa, Ethiopia

**DOI:** 10.1371/journal.pone.0299964

**Published:** 2024-10-10

**Authors:** Bethlehem Shawel, Yemane Berhane

**Affiliations:** 1 Reproductive Health and Population Department, Addis Continental Institute of Public Health, Addis Ababa, Ethiopia; 2 Epidemiology and Biostatistics Department, Addis Continental Institute of Public Health, Addis Ababa, Ethiopia; Belgrade University Faculty of Medicine, SERBIA

## Abstract

**Background:**

Anti-seizure medications (ASMs) are the primary therapeutic mode to control seizures in patients with epilepsy. Adherence to the medications is critical to achieving the goals of epilepsy therapy. However, the cost of the medications and the interrupted availability of ASMs contribute to non-adherence to epilepsy treatment. Therefore, this study aimed to assess ASM adherence and its association with self-reported medication availability and affordability.

**Objective:**

To assess whether self-reported availability and affordability of Anti-seizure medications affect medication adherence among Epileptic Patients at Eka Kotebe General Hospital, Addis Ababa, Ethiopia, from January 13, 2023 to March 23, 2023.

**Methods:**

A hospital-based analytical cross-sectional study was conducted among 357 epileptic patients using the Consecutive sampling method in Eka Kotebe General Hospital, Addis Ababa, Ethiopia. ASM adherence was measured using a self-report 3items questionnaire focusing on medication use patterns of patients from their last visit to the current visit. Statistical packages for Social Sciences 26.0 version statistical software cleaned, coded and analyzed the collected data. Binary logistic regression was fitted, and P-values less than 0.05 were considered to have statistical significance.

**Result:**

The prevalence of ASM adherence was 55.2% with 95% CI (50.1%; 60.2%). About two-thirds (61.3%) of patients in this study had limited access to the ASMs or could not afford the medications. Self-reported availability of ASMs (AOR = 2.01, 95% CI = 1.01, 3.98) was significantly associated with ASM adherence. Self-reported affordability of ASMs was associated with ASM adherence in the Bivariate logistic regression analysis; however, when adjusted for other covariates in the multivariable logistic regression, no significant association was observed (p = 0.674).

**Conclusion and recommendation:**

Only about half of the epileptic patients adhered to ASMs at Eka Kotebe General Hospital. Self-reported availability of ASMs was an essential factor. Improving access to ASMs is critical to improving adherence and management of epilepsy.

## Introduction

Medication adherence is the extent to which individuals take their medications as prescribed with respect to dosage and dosage intervals [1]. In both developed and developing countries, non-adherence to medication remains a significant concern for healthcare providers as well as patients because of its adverse consequences on therapeutic outcomes. It is significant, especially in chronic illnesses involving complex and long-term medical regimens [2].

Epilepsy is a common chronic neurological disease of the brain affecting 50 million people worldwide, of whom 40 million are estimated to live in developing countries [3]. Anti-seizure medications (ASMs) are the mainstay of long-term epilepsy treatment, and they are 70% effective at reducing epileptic attacks in adults [4]. However, about 90% of epileptic patients in developing countries are not receiving appropriate treatment due to cultural attitudes, lack of prioritization, poor healthcare system, and inadequate supply of anti-seizure medications (ASMs) [5].

In Ethiopia, epilepsy is often mistakenly seen as a form of mental illness by the Community and is usually treated by psychiatrists and psychiatric nurses. The most prescribed Anti-seizure medications are phenobarbitone, phenytoin, and sometimes carbamazepine and sodium Valproate.

A study in Western countries revealed an adherence rate of 71% in the US [6] and 59% in the UK [7]. The result was not different in Gondar, Ethiopia (70.8%) [8] and Jimma, Ethiopia (58.5%) [9]. On the other hand, the report was higher in India (98.6%) [10] and Palestine (85.3%) [11]. However, a lower adherence rate was found in China 51.9% [12], Nigeria (32.6%) [13], and South Africa (54.6%) [14].

The consequence of ASM nonadherence behavior has been associated with poor seizure control, increased morbidity and mortality along with increased time of hospitalization, worsened patient outcomes, poor quality of life, and increased healthcare cost. ASM nonadherence will also lead to an increase in the burden of inpatient and emergency department services; moreover, it also affects the family members socially, economically, and psychologically [15].

Availability and affordability of ASMs are poor and act as a barrier to accessing treatment for epilepsy in low and middle-income countries [16]. In most low-income countries, access to medicines remains very low. The availability and affordability of medications are two key factors that affect patients’ access to treatment. A study of the availability and prices of ASMs in southern Vietnam showed that only 57% of the public and private pharmacies surveyed had ASMs available [17]. A second study conducted in Zambia found that nearly one-half of the government, private, and nongovernmental organization (NGO) pharmacies surveyed did not carry ASMs [18]. There is a failure to explore these factors, particularly in Ethiopia.

A study done in Bangui, Central African Republic, on the Problem of accessibility to anti-seizure medications for patients who have epilepsy, shows that the lack of trained personnel, the inadequacy of pharmaceutical structures, the insufficient availability of anti-seizure medications, and their very high cost are factors limiting the accessibility of anti-seizure medications for epileptic patients [19].

This study aimed to evaluate medication availability and affordability from the patient’s point of view. This allows for a more direct assessment of the role of these factors in adherence. However, the studies and tools developed thus far were from a system perspective. Hence, this study aims to assess the association of self-reported medication availability and affordability with medication adherence. In addition, ASM adherence was also assessed to fill the gaps in epilepsy treatment.

## Methodology

### Study design and period

A hospital-based Analytical cross-sectional study with internal comparison was conducted from January 13, 2023 to March 23, 2023.

### Study setting

The study was conducted at Eka Kotebe General Hospital, located in the eastern part of Addis Ababa, the capital city of Ethiopia. Eka Kotebe General Hospital was established as an extension of Amanuel Mental Specialized Hospital until April 2020, when it became a stand-alone federal hospital. The hospital has a dedicated clinic for patients with epilepsy. There were about 2478 epileptic patients who had regular follow-ups in a year at the outpatient department, and on average, 200 epilepsy patients have follow-ups at the hospital. Patients pay for the services they get from the hospital and for purchasing ASMs, except those who use CBHI (community-based health insurance). The hospital also gives psychiatric services at the outpatient level. In addition, the hospital has served as a dedicated inpatient COVID treatment center.

### Source population

Adult epileptic patients who have been on treatment with one or more anti-seizure medications and who had follow-ups at the outpatient units during the study period.

### Study population

We selected adult epileptic patients who have been on treatment with one or more anti-seizure medications and who had follow-up at the outpatient units during the study period, fulfilling the inclusion criteria.

### Inclusion criteria

Adult Epileptic Patients (age≥18 years) who had a follow-up in the hospital for at least three months and received ASMs at the last visit were eligible for the study. Patients with general severe medical conditions and unable to communicate were excluded.

### Sample size and sampling technique

The sample size for the first objective was determined using a single population proportion formula considering the following assumptions (z = 1.96, d = 0.05, and P = 65%) [20]. Second, an attempt was made to calculate the sample size by considering the association of medication availability and affordability with adherence using Open Epi version 2.3.1. Finally, the sample size we obtained from the first objective was the largest and was taken as our final sample. By considering a non-response rate of 10%, the final estimated sample size was 385.

Consecutive sampling was used to recruit samples for the study each day of the data collection process until the desired sample size was obtained.

### Data collection tool and procedures

Data was collected using a structured questionnaire translated into a local language (Amharic) and pre-tested. Trained nurses interviewed volunteer participants face-to-face during their follow-up clinic visits. The data was collected after explaining the purpose of the study to the participants and earning informed written consent. The patient interview aimed to gather information related to sociodemographic, clinical factors, psychosocial factors, self-reported medication availability, affordability, and adherence. Their medical records were reviewed for the type of seizure and prescribed ASMs.

**Adherence** of epileptic patients to their ASMs was assessed using a self-reported questionnaire; in this study, a 3 items questionnaire focusing on medication use patterns of patients from their last visit to the current visit was used. Patients were asked 3questions: “Did you forget to take your medicine since your last visit?” “In the past two weeks, were there any days you did not take your medicine?” and “Did you take all your medicine yesterday?” The response choices were yes/no, and a response “no” was rated as “1,” and a “yes” response as “0,” except for item 3. For item 3, a response “yes” was rated as “1” and “no” as “0”. The total score ranges from 0 to 3, with scores of </ = 1, 1 to <3, and 3 reflecting low, medium, and high adherence, respectively. For data analysis, scores were categorized into two, scores <3 were assigned as non-adherent, and those who scored 3 were assigned as adherent.

### Data quality control

To ensure the data quality, a data collection tool was prepared after reviewing the literature related to the study. Data was collected by 4 BSC nurses who know about epilepsy and a supervisor. A thorough training was given by the principal investigator about the general objective of the study and the contents of the questionnaire. The questionnaire was pre-tested a week before the data collection started on 10% of the sample size at Eka Kotebe General Hospital Outpatient units. After a thorough and deep review of inputs obtained during the pre-test, the final tool was developed with some modifications. The pre-tested patients were excluded from the analysis. Data collectors were supervised daily, and the supervisor and principal investigator checked the filled questionnaires daily.

### Data analysis

The gathered quantitative data was cleaned, arranged, coded, and then analyzed through statistical packages for social sciences (SPSS) version 26.0 statistical Software. Categorical variables were expressed by percentage and frequency, whereas numerical variables were present by median with Inter Quartile range (IQR).

The association between the independent variables and Adherence to ASMs was analyzed using a binary logistic regression model. Bivariate binary logistic regression was run at a 25% significance level to screen out potentially significant independent variables. A multivariable binary logistic regression model was run by including the significant independent variables from the bivariate binary logistic regression model. To measure the presence and strength of association between the independent variables and adherence to ASMs, adjusted odds ratio (AOR), P-value, and 95% CI for AOR were calculated separately for self-reported availability and affordability of ASMs. Since the ORs were not different, in the final model, the two variables were considered together, and variables with a P-value of ≤ 0.05 were considered significantly associated with adherence to ASMs.

### Operational definitions

#### Adherence

In this study, adherence was measured using a self-report 3-item questionnaire focusing on medication use patterns of patients from their last visit to the current visit, the items were taken from Morisky Medication Adherence Scale-8.

#### Self-reported medication availability

In this study, medication availability was measured from the patient’s perspective based on their ability to obtain their medication in the same facility where treatment was given, a nearby facility, or near their residence. Accordingly, Medication Availability was assigned as “**Available**” if patients were able to gain medications in the same or nearby health facility where they received care or near their residency and others were categorized as “**Unavailable**”.

#### Self-reported medication affordability

In this study, medication affordability was assigned as “**Affordable**” if patients could pay for medications with no difficulty or gain medications free and others were categorized as “**Unaffordable**”.

#### ASM (Anti-seizure medications)

ASMs are the primary medications used to control seizures in epileptic patients. In the study setting, the most prescribed anti-seizure medications are phenobarbitone, phenytoin, and sometimes carbamazepine and sodium valproate. In this study, the number of ASMs used by patients was classified as monotherapy if participants took a single drug and poly-therapy, if the participants took more than one anti-seizure medication.

#### Social support

Social support is the physical and emotional comfort given to you by your family, friends, co-workers, and others. It is the knowledge that you are part of a community of people who love and care for you, value you, and think well of you. In this study, social support was assessed using a Likert scale with a single question: “Is someone available to help you if you need help?”.

#### Financial support

In this study, financial support was assessed using a single question: “Do you get financial support from relatives, organizations, or anybody else when needed?”.

### Ethical consideration

The research and ethical review board of the Addis Continental Institute of Public Health granted ethical clearance and approval of the study. Following the approval, Eka Kotebe General Hospital Institutional Review Board (IRB) obtained official permission. Before data collection, informed written consent was obtained from the study participants. Individuals were told that they had a right to withdraw from the study at any time, and this would not affect the service they got from the hospital. Confidentiality was ensured during the patient interviews and the review of charts.

## Results

### Socio-demographic characteristics

A total of 357 participants participated in the study, with a response rate of 92.7%. From this, 111 (31.1%) of the respondents were 25–34 years old. More than half of the study participants (61.1%) were single and 184 (51.5%) were males. Regarding educational status, the majority of the study participants had primary and secondary school, 95 (26.6%) and 109(30.5%), respectively. Around two-thirds of the study participants were unemployed, 191 (53.5%) were living in Addis Ababa, and nearly a third were within the same sub-city. The median (IQR) family size of the participants was 4 (3, 5) (Table 1).

**Table 1:** Socio-demographic characteristics of adult Epileptic patients in Eka Kotebe General Hospital, Ethiopia, 2023 (n = 357).

Variable	Category	Frequency	Percentage
**Family size (Median, IQR)**			4 (3,5)
**Age(Year)**	18–24	94	26.3
	25–34	111	31.1
	35–44	78	21.8
	≥ 45	74	20.7
**Sex**	Female	173	48.5
	Male	184	51.5
**Marital Status**	Single	218	61.1
	Married	139	38.9
**Educational Status**	No formal education	80	22.4
	Primary education (1–8)	95	26.6
	Secondary education (9–12)	109	30.5
	College and above	73	20.4
**Usual place of residence**	Within the same sub-city	109	30.5
	Addis Ababa	191	53.5
	Outside Addis Ababa	57	16
**Employment Status**	Gainfully Employed	130	36.4
	Unemployed	227	63.6
**Social Support**	Not often	87	24.4
	Sometimes	152	42.6
	Often	118	33.1
**Financial support**	No	181	71.5
	Yes	72	28.5

The majority of the respondents, 181(71.5%), had no financial support, and 118 (33.1%) thought they had someone around to help them often when they needed help. (Table 1).

### Clinical factors for anti-seizure medication adherence

Among the study participants, the majority of the patients, 279 (78.2%), were diagnosed with a Generalized type of seizure, and more than half, 227(63.6%) of the participants were on monotherapy. The median (IQR) age for the onset of the illness of the participants was 17(10, 24) years, and about slightly two-thirds, 223(62.5%), of the participants had a duration of illness of greater than/equal to 11 years. While co-morbidity was found in less than a fifth of the participants and a small number, 17(4.8%) of the participants had an adverse effect that prompted them to stop taking medication since their last visit (Table 2).

**Table 2:** Clinical factors for anti-seizure medication adherence among adult Epileptic patients at Eka Kotebe General Hospital, Ethiopia, 2023 (n = 357).

Variable	Category	Frequency	Percentage
**Age of onset of Epilepsy (Year) (Median, IQR)**			17(10,24)
**Duration of illness (in Years)**	<5 years	48	13.4
	6–10 years	82	23
	>11 years	223	62.5
**Types of Seizure**	Generalized	279	78.2
	Focal	66	18.5
	Unclassified	12	3.4
**Number of ASMs**	Monotherapy	227	63.6
	Polytherapy	130	36.4
**History of Adverse effects of ASMs that prompt to stop taking medication**	No	283	79.3
	Yes	74	20.7
**History of Adverse effects of ASMs that prompt to stop taking medication since the last visit**	No	340	95.2
	Yes	17	4.8
**Comorbidity**	No	308	86.3
	Yes	49	13.7

Comorbidity refers to a non-communicable disease that requires a long-term medication, such as Diabetes, hypertension, cardiac illness, renal disease, psychiatric disorders or other health conditions.

### Prevalence of adherence to ASMs and self-reported medication availability and affordability among adult Epileptic patients

The prevalence of ASM adherence was 55.2% with 95% CI (50.1%; 60.2%). In this study, self-reported availability and affordability of ASMs were found to be 80.1% and 53.2% respectively (Table 3).

**Table 3:** Prevalence of adherence to ASMs and self-reported medication availability and affordability among adult Epileptic patients at Eka Kotebe General Hospital, Ethiopia, 2023 (n = 357).

Variable	Category	Frequency	Percentage
**Adherence to ASMs**	Non-Adherent	160	44.8
	Adherent	197	55.2
**Self-reported Availability of ASMs**	Unavailable	71	19.9
	Available	286	80.1
**Self-reported Affordability of ASMs**	Unaffordable	167	46.8
	Affordable	190	53.2

**Fig 1:**
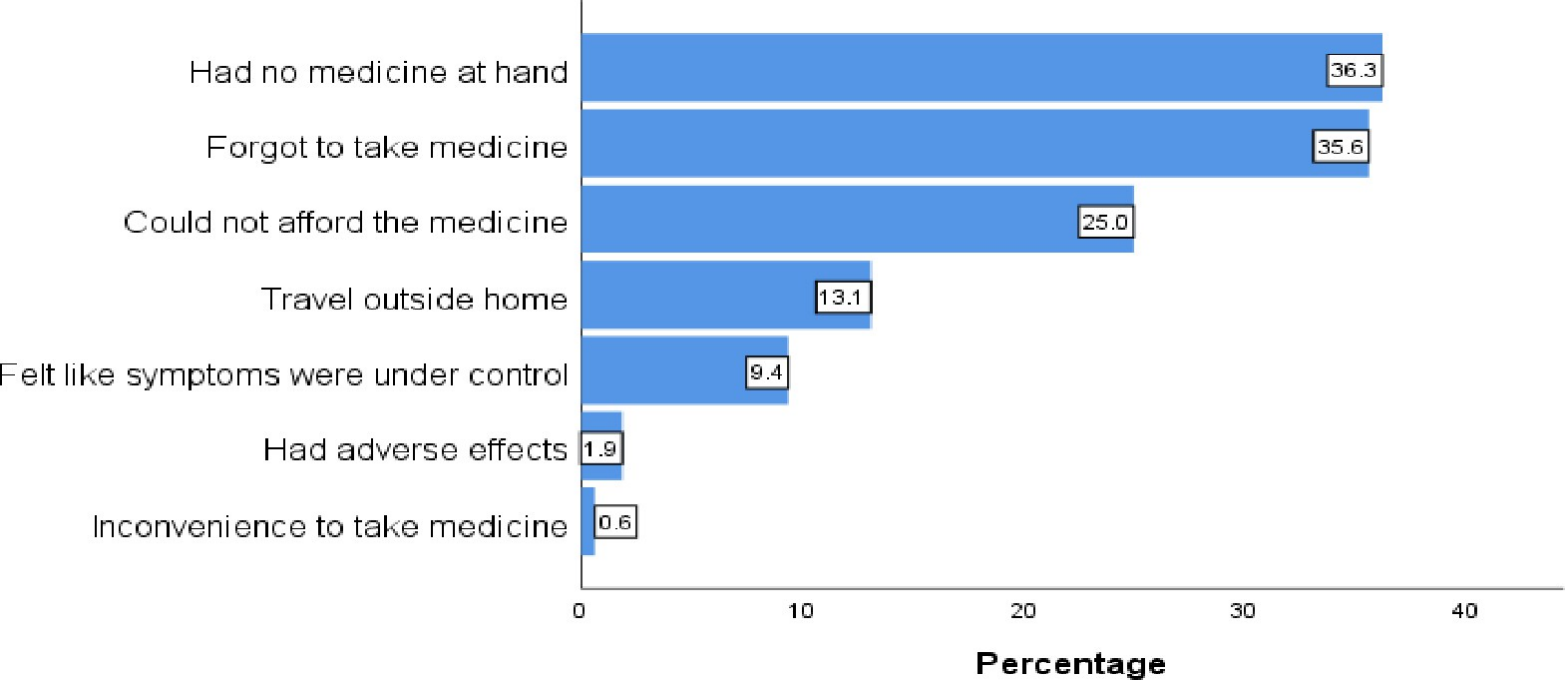
Reasons for ASMs non-adherence at Eka Kotebe General Hospital, Ethiopia, 2023 (n = 357).

The unavailability of the medications at hand 36.3% and forgetfulness to take medicine 35.6%, were the most common reasons for medication nonadherence, followed by medication unaffordability 25%, and travel outside the home 13.1% was the least reasons for medication non-adherence (Fig 1).

### Association of self-reported medication availability and affordability with anti-seizure medication adherence among people with epilepsy

In the bivariate binary logistic regression, educational status, number of ASMs, and self-reported affordability of ASMs were significantly associated with adherence to ASMs. However, on the multivariable binary logistic regression model, after adjusting for the covariates, Patients who finished secondary school, were educated up to college level and above, Number of ASMs, and self-reported Availability of ASMs were found to have a statistically significant relationship with adherence to ASMs at 5% level of significance.

Accordingly, after adjusting for other covariates, the odds of being adherent to ASMs among those with secondary education were 2.2 times higher as compared to those with no formal education (AOR = 2.21, 95% CI = 1.07, 4.56, p-value = 0.032). Similarly, the odds of adherent to ASMs were 2.6 times higher for those educated up to college level and above than those with no formal education (AOR = 2.61, 95% CI = 1.08, 6.30, p-value = 0.032). Regarding self-reported availability of ASMs, the odds of being adherent to ASMs were two times higher among those who reported the medications available as compared with those who reported unavailable (AOR = 2.01, 95% CI = 1.01,3.98, p-value = 0.046). On the other hand, the odds of being adherent to ASMs were 45% less for those on polytherapy than those on monotherapy (AOR = 0.55, 95% CI = 0.32, 0.96, p-value = 0.035). In bivariate logistic regression analysis, self-reported affordability of ASMs was associated with ASM adherence (COR = 1.59, CI = 1.04, 2.42, P-Value = 0.031); however, when adjusted for other covariates in the multivariable logistic regression, no significant association was observed (p = 0.674) (Table 4).

**Table 4:** Bivariate and multivariate analysis of variables associated with ASM adherence (n = 357).

Variables	Category	Adherence to ASMs		COR (95% CI)	P-Value	AOR (95% CI)	P-Value
		Adherent (n)	Non-adherent (n)				
**Age (years)**	18–24	53	41	1		1	
	25–34	60	51	0.91 (0.52–1.58)	0.738	1.14(0.55–2.35)	0.729
	35–44	43	35	0.95 (0.52–1.74)	0.869	0.99(0.46–2.13)	0.969
	≥ 45	41	33	0.96 (0.52–1.78)	0.899	0.95 (0.42–2.18)	0.910
**Sex**	Female	96	77	1		1	
	Male	101	83	0.98(0.64–1.48)	0.909	0.88(0.52–1.49)	0.637
**Educational Status**	No formal education	37	43	1		1	
	Primary education (1–8)	45	50	1.05(0.58–1.90)	0.883	1.10 (0.53–2.30)	0.795
	Secondary education (9–12)	68	41	**1.93(1.07–3.46)**	**0.028**	**2.21 (1.07–4.56)**	**0.032** *
	College and above	47	26	**2.10(1.10–4.03)**	**0.025**	**2.61 (1.08–6.30)**	**0.032** *
**Number of Anti-seizure medications**	Monotherapy	138	89	1		1	
	Polytherapy	59	71	**0.54(0.35–0.83)**	**0.005**	**0.55 (0.32–0.96)**	**0.035** *
**Comorbidity**	No	171	137	1		1	
	Yes	26	23	0.91(0.50–1.66)	0.748	0.78 (0.34–1.80)	0.557
**Self-reported Availability of ASMs**	Unavailable	35	36	1		1	
	Available	162	124	1.34 (0.80–2.26)	0.266	**2.01 (1.01–3.98)**	**0.046** *
**Self-reported Affordability of ASMs**	Unaffordable	82	85	1		1	
	Affordable	115	75	1.59(1.04–2.42)	**0.031**	1.13 (0.64–2.01)	0.674
**Financial Support**	No	92	89	1		1	
	Yes	38	34	1.08(0.63–1.87)	0.780	0.93 (0.52–1.68)	0.819

* Statistically Significant at P<0.05

## Discussion

Findings of adherence in this study showed that less than two-thirds (55.2%; 95% CI (50.1%-60.2%) of the study participants were adherent to anti-seizure medications. Globally, the adherence rate ranges between 33 and 99% [6, 8, 10, 12, 13].

The prevalence of adherence to anti-seizure medications in this study was in line with a cross-sectional study conducted in Addis Ababa (52.4%) [21], Jimma (58.5%) [9], and Nigeria (55.5%) [22]. However, it was lower than the study done in Gondar, Ethiopia (61.5%) [23], Southwestern Ethiopia (63.5%) [24], and India (72.3%) [25]. The probable reason for these discrepancies could be the differences in the methodology used for assessing adherence rate and the study design. For example, a prospective observational study was employed in the study from southwestern Ethiopia, and adherence was assessed by the Hill–Bone compliance to the high blood pressure therapy scale [24], but the current study employed a cross-sectional study design, and adherence was assessed using self-report questions taken from a validated MMAS 8. The other reason could be due to the socio-demographic characteristics of the study participants as well as the study area.

On the other hand, the findings of this study were higher than the studies done in Brazil and Nigeria, which were (33.8%) [26] and (31.6%) [13], respectively, and this difference was probably due to the difference in ASMs multidrug treatment. For instance, 71.1% of people with epilepsy in Brazil and 85% in Nigeria were on multiple ASM treatment, while in the current study, only 36.4% of people with epilepsy were in poly-ASM treatment.

The most common reasons for missing doses in this study were the unavailability of the medications at hand (36.3%) followed by forgetfulness to take medicine (35.6%), which is supported by the findings of studies in Amanuel Mental Specialized Hospital, Ethiopia [27], Jimma Ethiopia [24] and Nigeria [22].

In this study, factors found to have a significant association with adherence to ASMs were higher educational status, self-reported availability of ASMs, and being on poly-therapy. This study revealed the odds of being adherent to ASMs were 2.6 times higher for those who were educated up to college level and above than those with no formal education. This result aligns with the study conducted in India [28]. This might be due to patients who are more educated might question their health care providers about their disease and its medications, might be more aware of the disease progression and follow the prescription and instructions as told Whereas, those who are less educated in our setting might prefer other alternative traditional practices at home than proper medication follow up.

Besides, Patients taking combination drugs were less likely to Adhere. Poly-therapy increases the potential for drug-drug interactions, results in failure to evaluate the individual drugs may affect compliance and is associated with a higher cost of medication and the necessity for therapeutic drug monitoring [29]. Our study revealed the odds of being adherent to ASMs were 45% less for those on poly-therapy as compared with those who were on monotherapy. Similar studies found a negative association between the complexity of medication and patient adherence [22, 28]. Regarding self-reported availability of ASMs, the odds of being adherent to ASMs were two times higher among those who reported the medications available as compared with those who reported unavailable. This finding was supported by the study from Jimma, Ethiopia [24], and India [28].

In bivariate logistic regression analysis, self-reported affordability of ASMs was associated with ASM adherence. This finding was supported by a study from South Western Ethiopia [30]. The possible justification was that patients who got their medication out of pocket would sometimes cease to purchase their medications, and those who got free will continue their medications in whatever circumstances.

By contrast, the patient’s adherence to their medication did not show a significant association with age and gender. This result is in line with the findings in Gondar, Ethiopia [23], Nigeria [22], and India [28]. This may indicate that ASM adherence in our context is affected more by the availability of the drugs rather than the demographic characteristics of the patients.

This study has a few limitations. First, it was a cross-sectional study with no prospective follow-up period. As a result, it may be subjected to recall bias. Second, a self-reported measure of adherence, which is prone to social desirability bias, was used to measure adherence. Third, it was conducted in a single setting, which makes it difficult to generalize.

## Conclusion and recommendation

In conclusion, overall, only about half of the patients adhered to Anti-seizure medications at Eka Kotebe General Hospital. The self-reported availability of ASMs was significantly associated with adherence to anti-seizure medications. Improving access to ASMs is critical to improving adherence and management of epilepsy. A large-scale study is highly recommended to fully understand the factors contributing to ASM adherence in Ethiopia.

## Supporting information

S1 File English version of questionnaires. (PDF)

S2 File Amharic version of questionnaires. (PDF)

## Abbreviations

AOR: Adjusted Odds Ratio
ASMs: Anti-Seizure Medications
CBHI: Community-Based Health Insurance
CI: Confidence Interval
COR: Crudes Odds Ratio
IQR: Inter Quartile Range
IRB: Institutional Review Board
OR: Odds ratio
SPSS: Statistical Package for Social Sciences
