# Supplementary material for: Adherence to anti-seizure medications and self-reported availability and affordability of the medications in Addis Ababa, Ethiopia
Source: PLoS One. 2024 Oct 10;19(10):e0299964. doi: 10.1371/journal.pone.0299964 (PMC11469612; doi:10.1371/journal.pone.0299964)
Supplement: S1 File — (PDF) [file pone.0299964.s001.pdf]

Annex II: English Version of Questionnaires

Eka Kotebe General Hospital, Addis Ababa, Ethiopia			
**Part I: Socio-demographic Characteristics**			
**No.**	**Question**	**Response and coding**	**Remarks**
1.	Age (in complete years)	[___|___]	
2.	Sex of respondent	1. Female<br>2. Male	
3.	Marital status	1. Single<br>2. Married<br>3. Divorced/separated<br>4. Widowed	
4.	Level of education	1. No formal education<br>2. Primary education (1-8)<br>3. Secondary education (9-12)<br>4. Vocational certificate<br>5. Diploma<br>6. Degree and higher	
5.	Where is your usual place of residence?	1. Within the Same sub city<br>2. Addis Ababa<br>3. Outside of Addis Ababa	
6.	How many people live together currently in your household?	[___|___]	
7.	What is your employment status?	1. Gainfully Employed<br>2. Not gainfully employed<br>3. Student<br>4. Unemployed	Skip to Part II, if the answer is 'Not gainfully employed' to this question.
8.	On average, how much do you earn from your Occupation in a month?	_________Birr	
**Part II: Clinical Characteristics**			
9.	What was your age when you were first diagnosed with epilepsy?	[___|___]	
10.	Type of seizure	1. Generalized<br>2. Complex partial<br>3. Others	

11.	Number of antiepileptic drugs	1. None<br>2. Mono therapy<br>3. Di therapy<br>4. Poly therapy	
12.	Have you ever experienced the adverse effect of the drugs (For example Depressed mood, confusion, weakness, blurring of vision, headache, nightmare, forgetfulness, skin rash, irritability and others) that prompted you to stop taking them?	1. Yes<br>2. No	
13.	Have you experienced the adverse effect of the drugs (For example Depressed mood, confusion, weakness, blurring of vision, headache, nightmare, forgetfulness, skin rash, irritability and others) that prompted you to stop taking them since your last visit to this clinic?	1. Yes<br>2. No	
14.	Do you have any co-morbidity that requires long-term medication other than epilepsy? (For example Diabetes, hypertension, cardiac illness, renal disease, psychiatric disorders or others…)	1. Yes<br>2. No	
**Part III: psychosocial Characteristics (social support)**			
15.	Is someone available to help you if you needed help?	1. Very often<br>2. Often<br>3. Sometimes<br>4. Not often<br>5. Never	
**Part IV: AEDs Availability and Affordability**			
16.	Where did you obtain your last AEDs?	1. In the same pharmacy/facility where you get treatment<br>**2.** In a nearby pharmacy/facility either where you get treatment or to your residence<br>**3.** From a pharmacy/facility neither close	

		to this facility nor to my home	
17.	Who pays for your anti-epileptic drugs often?	1. Self<br>2. Family/Relative<br>3. Receiving free medication<br>4. Insurance<br>**5.** Employer organization	
18.	Did you have any financial challenges to get your medications for the last prescription?	1. No<br>2. Yes	
19.	Have you ever had financial challenges to get your medications in the past?	1. No<br>2. Yes	Skip to Part V, if 'No' to this question.
20.	Do you get financial support from relatives, organizations, or anybody else when needed?	1. Never<br>2. Not now<br>3. Yes, I receive financial support	
**Part V: Assessment of adherence**			
	**Questions: refers to the period between the last visit and the current visit.**	**Yes**	**No**
1.1.	Did you forget to take your medicine since your last visit?		
1.2.	In the past two weeks, were there any days you did not take your medicine?		
1.3.	Did you take all your medicine yesterday?		
1.4.	If you missed your medicine even once since the last visit, what was the reason:	**1.** Travel or slept outside home<br>2. Because I felt like symptoms were under control<br>3. Had adverse effects<br>4. Because of the inconvenience to take medicine<br>5. Forgot to take medicine<br>6. Had no medicine at hand<br>7. Could not afford the medicine<br>8. Other	
