# Supplementary material for: Adherence to anti-seizure medications and self-reported availability and affordability of the medications in Addis Ababa, Ethiopia
Source: PLoS One. 2024 Oct 10;19(10):e0299964. doi: 10.1371/journal.pone.0299964 (PMC11469612; doi:10.1371/journal.pone.0299964)
Supplement: S2 File — (PDF) [file pone.0299964.s002.pdf]

Annex IV: የአማርኛ መጠይቅ ቅፅ

**ኤካ ኮተቤ አጠቃላይ ሆስፒታል, አዲስ አበባ ,ኢትዮጵያ**			
**ክፍል 1. ስለ ታካሚው አጠቃላይ መገለጫዎች**			
**ተ.ቁ**	**ጥያቄዎች**	**መልሶች እና ኮድ መስጠት**	**አስተያየቶች**
1.	እድሜህ/ሽ ስንት ነው (በቁጥር ይፃፍ)	[___|___]	
2.	ጾታ	1. ሴት<br>2. ወንድ	
3.	የጋብቻ ሁኔታ	1. ያላገባ<br>2. ያገባ<br>3. የፈታ/ች<br>4. ባል የሞተባት /ሚስት የሞተችበት	
4.	የትምህርት ደረጃ	1. መደበኛ ትምህርት የለም<br>2. አንደኛ ደረጃ (ከ 1-8 ክፍል)<br>3. ሁለተኛ ደረጃ (ከ9-12 ክፍል)<br>4. የሙያ የምስክር ወረቀት<br>5. ዲፕሎማ<br>6. ዲግሪ እና ከዛ በላይ	
5.	አዘወትረው የሚኖሩበት የመኖሪያ ቦታዎ የት ነው?	1. በዚሁ ክፍለ ከተማ ውስጥ<br>2. አዲስ አበባ<br>3. ከአዲስ አበባ ውጪ	
6.	በአሁኑ ጊዜ በእርስዎ ቤተሰብ ውስጥ ስንት ሰዎች አብረው ይኖራሉ?	[___|___]	
7.	የስራ ሁኔታህ/ሽ ምንድን ነው?	1. ገቢ ባለው መልኩ ተቀጥ ረው ይሰራሉ<br>2. ገቢ የሌለው ስራ ይሰራሉ<br>3. ተማሪ<br>4. ምንም ስራ አልስራም	ለዚህ ጥያቄ ‘ገቢ የሌለው ስራ እሰራለው’ ከሆነ መልሰ ወደ ክፍል 2 ይዝለሉ።

8.	በአማካኝ ወርሃዊ ገቢዎ ምን ያህል ነው?	________________ብር	
**ክፍል 2: ክሊኒካዊ ባህሪያት**			
9.	የሚጥል በሽታ እንዳለብህ/ሽ ሲታወቅ ዕድሜህ/ሽ ስንት ነበር?	[___|___]	
10.	የማንቀጥቀጥ አይነቶች	1. ሙሉ የሰውነት አካል<br>2. ግማሽ የሰውነት ክፍል<br>3. ሌላ	
11.	የሚወሰዱ መድሃኒቶች ብዛት	1. ምንም<br>2. አንድ መድሃኒት<br>3. ሁለት መድሐኒት<br>4. ብዙ መድሃኒት	
12.	መድሃኒቱን መውሰድ እንዲያቆሙ የሚገፋፋዎት የጎንዮሽ ጉዳ ት አጋጥሞዎት ያውቃል? (ለምሳሌ የመንፈስ ጭንቀት፣ ግራ መጋባት፣ ድክመት፣ የዓይን ብዥታ፣ ራስ ምታት፣ ቅዠት፣ መርሳት፣ የቆዳ ሽፍታ፣ መነጫነጭ እና ሌሎችም)	1. አ ዎ<br>2. አይ	
13.	ወደዚህ የህክምና ተቋም ለመጨረሻ ጊዜ ከጎበኙ በኋላ መድሃኒቱን መውሰድ እንዲያቆሙ የሚገፋፋዎት የጎንዮሽ ጉዳት አጋጥሞዎታል? (ለምሳሌ የመንፈስ ጭንቀት፣ ግራ መጋባት፣ ድክመት፣ የዓይን ብዥታ፣ ራስ ምታት፣ ቅዠት፣ መርሳት፣ የቆዳ ሽፍታ፣ መነጫነጭ እና ሌሎችም)	1. አ ዎ<br>2. አይ	
14.	ከሚጥል በሽታው በተጨማሪ የረጅም ጊዜ መድሃኒት የሚያስፈልገው በሽታ አሎት? (ለምሳሌ የስኳር በሽታ፣ የደም ግፊት፣ የልብ ሕመም፣ የኩላሊት በሽታ፣ የአእምሮ ሕመም ወይም ሌሎች...)	1. አ ዎ<br>2. አይ	
**ክፍል 3፡ ሳይኮሶሻል ባህርያት (ማህበራዊ ድጋፍ)**			
15.	እርዳታ በምትፈልግበት/በምትፈልጊበት ሰዓት አቅርቦ የሚያወራህ ሰው ታገኛለህ/ሽ	1. በጣም ብዙ ጊዜ<br>2. ብዙ ጊዜ	

		3. አንዳንድ ጊዜ<br>4. በጥቂቱ<br>5. በፍጹም	
**ክፍል 4: የሚጥል በሽታ መድኃኒት ተገኝነት እና የዋጋ ተመጣጣኝነት**			
16.	የመጨረሻ የህክምና ክትትሎ ጊዜ የታዘዘሎትን የሚጥል በሽታ መድኃኒት ከየት ነው የገዙት?	1. ህክምና በሚያገኙበት በተመሳሳይ ፋርማሲ/ ተቋም ውስጥ<br>2. ህክምና በሚያገኙበት ወይም ወደ መኖሪያዎ አቅራቢያ በሚገኝ ፋርማሲ/ የህክምና ተቋም ውስጥ<br>3. ህክምና በሚያገኙበት ወይም ወደ መኖሪያዎ አቅራቢያ ባልሆነ ፋርማሲ/ ተቋም ውስጥ	
17.	የሚጥል በሽታ መዳኒቶን ለመግዛት በአብዛኛው ጊዜ ማን ይከፍልሎታል?	1. ራሴ<br>2. ቤተሰብ/ዘመዶች<br>3. ነፃ መድሃኒት መቀበል<br>4. ኢንሹራንስ<br>5. የምሰራበት ድርጅት	
18.	የመጨረሻ የህክምና ክትትሎ ጊዜ የታዘዘሎትን የሚጥል በሽታ መድኃኒት ለማግኘት የገንዘብ ችግር አጋጥሞዎታል?	1. አይ<br>2. አ ዎ	
19.	ከዚህ ቀደም መድሃኒቶችዎን ለማግኘት የገንዘብ ችግሮች አጋጥመውዎት ያውቃሉ?	1. አይ<br>2. አ ዎ	ለዚህ ጥያቄ ‘አይ’ ከሆነ መልሶ ወደ

			ክፍል 4 ይዝለሉ።
20.	በሚያስፈልግበት ጊዜ ከዘመዶች፣ ድርጅቶች ወይም ከማንም ሰው የገንዘብ ድጋፍ ታገኛለህ/ሽ?	1. በጭራሽ<br>2. አሁን አላገኝም<br>3. አዎ፣ የገንዘብ ድጋፍ አገኛለሁ	
**ክፍል 5: መድኃኒትን በታዘዘው መሰረት በአግባቡ ስለመውሰድ**			
	ጥያቄዎች፡ በመጨረሻው የህክምና ክትትል ጉብኝት እና አሁን ባለው ጉብኝት መካከል ያለውን ጊዜ ያመለክታል።	አ ዎ	አይ
1.1.	ከመጨረሻው የህክምና ክትትል ጉብኝትዎ ጀምሮ መድሃኒትዎን መውሰድ ረስተዋል?		
1.2.	ባለፉት ሁለት ሳምንታት መድሃኒትዎን ያልወሰዱባቸው ቀናት ነበሩ?		
1.3.	ትላንትና ሁሉንም መድሃኒት ወስደዋል?		
1.4.	ካለፈው የህክምና ክትትል ጉብኝትዎ በኋላ አንድ ጊዜ እንኳን መድሃኒትዎን ካልወሰዱ ምክንያቱ ምን ነበር፡-	1.በጉዞ ምክንያት ወይም ከቤት ውጭ አድረው<br>2. ህመሙ የተሻሉ (የህመሙ ስሜቶች መጥፋት) ስለተሰማዎ<br>3. የመድኃኒቶቹ የጎንዮሽ ጉዳት ስለነበሮ<br>4. መድሃኒት መውሰድ ምቾት ስለማይሰጡ<br>5. መድሃኒት መውሰድ ረስተው<br>6. በእጅ ምንም መድሃኒት አልነበረውም<br>7. መድሃኒቱን መግዛት አልቻሉም<br>8. በሌላ በምክንያት	
